# Mutations in two global regulators lower individual mortality in *Escherichia coli*

**DOI:** 10.1111/j.1365-2958.2007.05988.x

**Published:** 2007-11-25

**Authors:** Fanette Fontaine, Eric J Stewart, Ariel B Lindner, François Taddei

**Affiliations:** 1 Inserm, U571 Paris, F-75015, France.; 2 Université Pierre et Marie Curie Paris, F-75005, France.; 3 Université Paris Descartes Fac Med Necker, Paris, F-75730, France.

## Abstract

There has been considerable investigation into the survival of bacterial cells under stress conditions, but little is known about the causes of mortality in the absence of exogenous stress. That there is a basal frequency of cell death in such populations may reflect that it is either impossible to avoid all lethal events, or alternatively, that it is too costly. Here, through a genetic screen in the model organism *Escherichia coli*, we identify two mutants with lower frequencies of mortality: *rssB* and *fliA*. Intriguingly, these two genes both affect the levels of different sigma factors within the cell. The *rssB* mutant displays enhanced resistance to multiple external stresses, possibly indicating that the cell gains its increased vitality through elevated resistance to spontaneous, endogenous stresses. The loss of *fliA* does not result in elevated stress resistance; rather, its survival is apparently due to a decreased physical stress linked to the insertion of the flagellum through the membrane and energy saved through the loss of the motor proteins. The identification of these two mutants implies that reducing mortality is not impossible; rather, due to its cost, it is subject to trade-offs with other traits that contribute to the competitive success of the organism.

## Introduction

Understanding the mechanisms underlying the survival of bacterial cells, defined as the capacity of the cell to maintain its integrity and undertake division (Barer and Harwood, 1999), may aid in the successful control of the growth and persistence of these organisms. Survival and death of the model organism *Escherichia coli* have been extensively studied on cells exposed to external sources of stress, such as high temperature, pH, UV, oxidative agents, antibiotics, high salt concentration, heavy metals and starvation (Storz and Hengge-Aronis, 1999; and references therein). Those studies have increased the understanding of how these external stresses injure cells and lead to the identification of several stress-response systems involved in the survival of bacterial cells. The nature of these pathways varies from very specific, such as the case of heavy metal resistance, where only a few genes are involved, to very general, such as the RpoS stress-response system, where more than 80 genes are co-ordinately regulated in response to a variety of stresses (Weber *et al*., 2005).

However, even in the absence of identifiable exogenous stress, there remains a measurable, basal death frequency in *E. coli* populations. This frequency in growing cells has been reported to be between 1 in 400 cells in plating experiments (Gallant and Palmer, 1979) and 1 in 2000 in exponentially growing populations on a solid surface (Stewart *et al*., 2005). In 1-day-old colonies, the frequency has been measured to be about 3 in 100 (Saint-Ruf *et al*., 2004). One explanation for the cause of death in these cells may be aging of the cells through multiple divisions. However, while an association has been found between the dead cells in an exponentially growing microcolony and their age measured by the age of the old pole in the population lineage, the age of the cell apparently plays only a small role in contributing to the death of these cells (Stewart *et al*., 2005). Therefore, it is unlikely that cell aging is the only factor responsible for the observed death frequency. There are other potential causes of death, in the absence of obvious external stress, which could be due to stochastic events within the cell. One candidate for such events is naturally occurring genetic mutations; however, the expected rate of lethal events, calculated at 1 in 20 000 cell divisions, is too rare to account for the observed frequencies (Kibota and Lynch, 1996). A second possibility is the occurrence or accumulation of protein errors that arise during the process of transcription and translation. These processes have been estimated to result in the miss-incorporation of an amino acid in up to 22% of the proteins synthesized in *E. coli* (Kurland *et al*., 1996). However, attempts to verify that such errors can lead to cell death by measuring cell mortality in mutants with altered levels of protein synthesis fidelity failed to show a significant effect (Jörgensen and Kurland, 1987).

While it is known that oxidative damage is a major cause of loss of cultivability during nutrient depletion (48 h culture in rich media), it has proven difficult to predict what processes in the cell are involved in the basal mortality in these cells in growing and early stationary-phase cultures (Cuny *et al*., 2005; Fredriksson and Nystrom, 2006). In this study, we have undertaken a genetic screen to identify mutants with an altered frequency of cell death in absence of exogenous stress.

As mutants with elevated mortality may potentially occur in any essential cell process, they should reflect potential causes of cell death that are normally avoided by the wild-type (WT) cell. As in the parallel case of studying mutants that alter the genetic mutation frequency, it is the antimutators, or in our case, the antimortality mutants that will be informative concerning the causes of spontaneous events (Sargentini and Smith, 1985). Thus, we focused on identifying mutants that exhibit lowered death frequencies (antimortality mutants), as these mutants are likely to be involved in processes related to the causes of spontaneous cell death, and hence may be useful for understanding these causes. Here, by a microscopy-based screen for death on the single-cell level, we identify two genes, *rssB* and *fliA*, which, when inactivated, result in lower frequencies of death. The products of these genes are involved in the regulation of major genetic networks. We investigate their role in cell death, their costs and benefits, and the potential contribution of resource competition to cell fate.

## Results

### Identification and verification of altered death frequency mutants

**Fig. 1:**
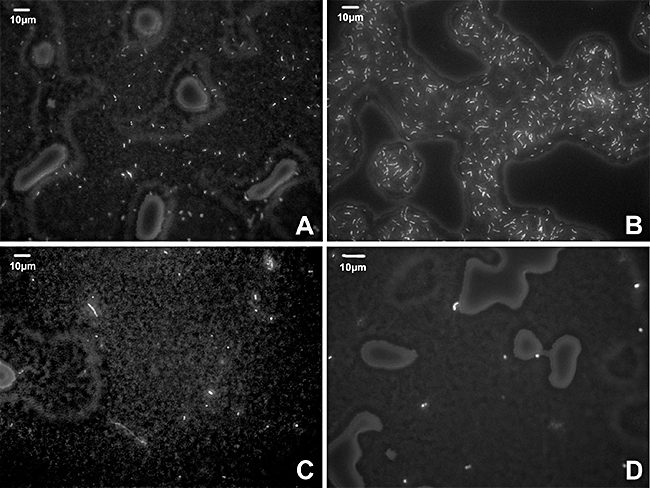
Qualitative screen for mutants with fewer dead cells. Cultures of the mutants, WT and controls were spotted onto agar plates containing PI, and screened visually in the microscope. Images are 40× magnification grey-scale pictures taken with simultaneous phase contrast and fluorescent light. Bright cells represent dead cells stained with PI; mutants with altered numbers of dead cells are identifiable by this method. A. WT control strain. B. Control *rpoS* mutant showing increased cell death. C. Decreased death mutant *fliA*. D. Decreased death mutant *rssB*.

We have developed a microscopy-based, qualitative screen to identify mutants of *E. coli* with altered death frequencies. Three thousand isolates of an ordered transposon insertion library were screened for differential rates of propidium iodide (PI)-stained cells in patches of cells grown to early stationary phase on solid medium. PI is a fluorescent dye which can only enter cells with damaged membranes, and hence can be used as a marker for some types of cell death (Lopez-Amoros *et al*., 1995). While it is possible to identify isolates with both greater and lesser rates of PI-stained cells, it was expected that identification of mutants with reduced death frequencies would be more revealing of the causes and mechanisms of cell death in WT cells. The initial screen was performed on cultures that had grown to early stationary phase at 42°C. These conditions were chosen to increase the chances of finding candidate mutations by augmenting the difference compared with WT. All candidates with apparently reduced rates of PI staining in the microscopic screening procedure were isolated for further studies (Fig. 1 and Table S1), where they were characterized under additional conditions, including the absence of external stress (described below). The phenotypes of these mutants were verified to be linked to the Tn10 insertions by co-transduction (there is co-transduction between the phenotype and the Tn10 insertion in the 100 of 100 tested transductants).

**Fig. 2:**
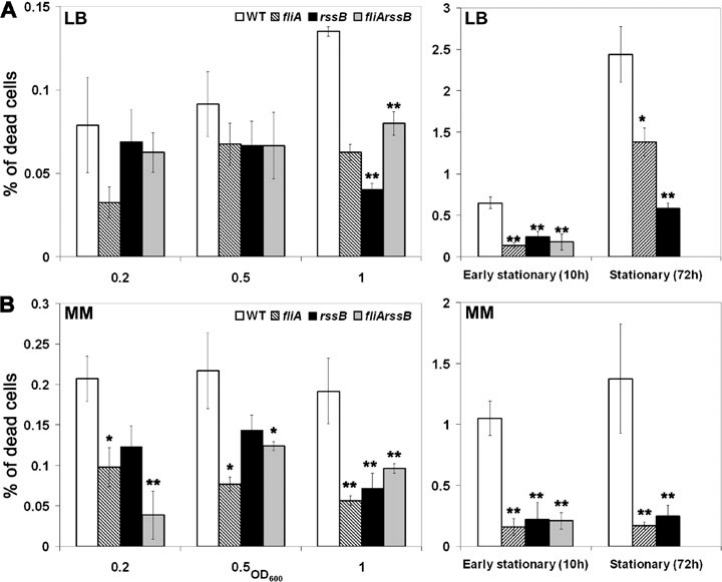
Quantification of dead cells in liquid cultures by FC analysis. Measurements were performed on WT (white bars), *fliA* (striped bars), *rssB* (black bars) and *fliArssB* (grey bars) cells grown at 30°C, on LB (A) or in Minimal medium (B) stained with SYTOX Green. Values correspond to 5–20 independent repeats. Error bars represent the standard error. The asterisk corresponds to *P*-values between < 0.05 and < 0.001, relative to WT by unpaired t-test. The double asterisks correspond to *P*-values < 0.001, relative to WT by unpaired *t*-test.

These 10 candidate mutants were analysed by flow cytometry (FC) to quantify the proportion of dead cells in comparison with WT cultures. To label dead cells and screen out mutants specifically sensitive to PI staining, an alternative dead cell stain, SYTOX Green (SG), was used (Roth *et al*., 1997). Liquid cultures of the strains in different growth phases were quantified for their fraction of fluorescently stained cells by FC. Of the 10 candidates, two isolates, containing insertions in the *rssB* and *fliA* genes, were confirmed to have a significantly reduced frequency of stained cells compared with WT in both Luria–Bertani (LB) and M9 media (Table S1). Construction of complete gene deletions of *rssB* and *fliA* confirmed that the phenotypes of Tn10 insertion mutants are due to the inactivation of the named genes. *fliA* cultures have significantly fewer dead cells than the WT in every phase of growth, except in early exponential phase in rich media, while for *rssB*, results are significant from OD_600_ = 1 (Fig. 2). The differences between WT and mutant strains remain constant in long-term cultures (up to 4 days, data not shown). The proportion of dead cells in the double mutant strain *fliArssB* is not different from the single mutant *fliA* in every condition (Fig. 2).

Use of Sytox green and PI are known to underestimate the number of dead cells (Lebaron *et al*., 1998; Ericsson *et al*., 2000), and refer only to one definition of death in bacteria, specifically the loss of membrane permeability. To verify the FC results, we used an alternative criterion for death: the absence of growth of single cells. This direct count of viable cells may overestimate the number of dead cells, in that it counts cells that may be alive but not growing during the time of observation. The combination of this method with the results from dead cell staining puts upper and lower limits on the dead cell count. Samples from the analysed cultures were grown on M9-agarose media in a microscope cavity slide, and incubated 4 h at 30°C. The proportion of cells that fail to form microcolonies on these slides is similar to that obtained by FC (in early stationary phase: WT 1.96%; *rssB* 0.52%, *P* = 0.003; *fliA* 0.43%, *P* < 0.001; *P*-values relative to WT by χ^2^-test).

Therefore, we show, using two different measures of cell death (cells with permeable membranes, and cells unable to grow and divide), that *rssB* and *fliA* mutant strains manifest fewer dead cells than WT during early stationary phase in both M9 and LB media. We cannot formally exclude the hypothesis that these mutants have the same or greater proportion of dead cells, but that these are masked due to immediate lysis of these cells; however, observation of growing microcolonies under the microscope (data not shown) did not indicate a higher frequency of lysed cells. In addition, it is unlikely that dead cells observed in exponential phase come from the initial inoculate. In fact, the dilution factor is such that the number of cells transferred in the initial inoculum is much lower than the number of dead cells observed later. Furthermore, increasing this dilution factor by 10-fold resulted in no difference in the number of dead cells counted later (data not shown). Therefore, dead cells present at the start of the subculture do not significantly contribute to the observed results.

### The phenotypes of the mutants manifest through known pathways

As RssB and FliA are involved in the regulation of major genetic networks, other genes in the same pathways were analysed to determine their frequencies of dead cells. The experiments described below are performed in early stationary phase, where we have the strongest differential signal between the mutants and the WT strain.

**Fig. 3:**
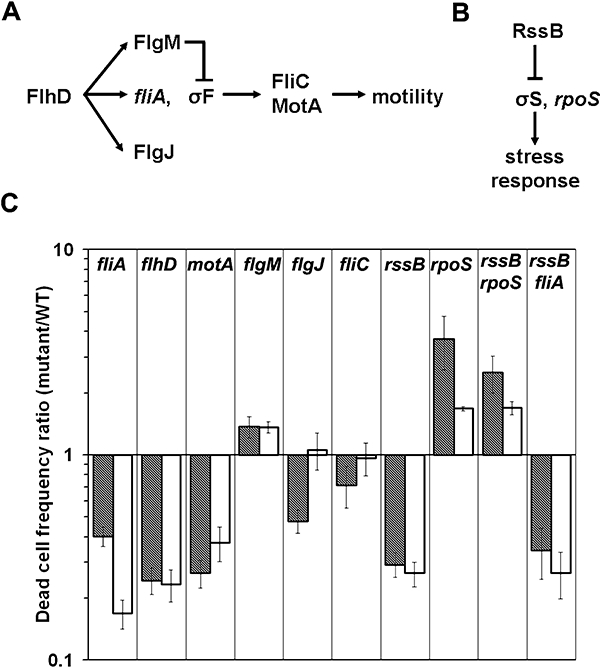
Gene regulation pathways and quantification of dead cells. A. Simplified schematic of the motility regulon. B. The general stress-response regulon. For both schematics, arrows indicate a positive effect, while blocked lines indicate a negative effect. C. Quantification of dead cells by FC analysis. Measurements were performed on early stationary-phase cultures grown at 30°C, stained with SYTOX Green. White bars represent cultures grown in M9; striped bars represent cultures grown in LB. The values reported are the ratio of the fraction of dead cells in the mutant strain compared with the fraction of dead cells in WT. Each bar represents mean of 5–15 repeats, each in turn consisting of three parallel cultures. The total number of cells assayed per condition is at least 3 × 10^6^. The only strains not significantly different from WT are *fliC* and *flgJ* (*fliC* in LB *P* = 0.416; in M9 *P* = 0.342; *flgJ* in M9, *P* = 0.926).

The product of the *fliA* gene is the sigma factor responsible for the transcription of much of the flagellar and chemotaxis genes (Fig. 3A) (Macnab, 1996). Expression of the *fliA* gene is activated by FlhD, and the activity of the *fliA* gene product, σ^F^, is negatively regulated by FlgM, an anti-sigma factor (Kutsukake, 1997). Mutants of *flhD* and *flgM* were therefore tested for their effects on the frequency of dead cells. Compared with WT, the *flhD* mutant has reduced levels of dead cells, with levels similar to *fliA*. The *flgM* mutant has a small, but significant increase in dead cell frequencies relative to WT, consistent with its antagonistic role in the activity of FliA (Kutsukake and Iino, 1994; Chilcott and Hughes, 2000) (Fig. 3).

The product of the *rssB* gene is involved in regulating the proteolysis of the RpoS sigma factor (σ^S^), which controls the expression of the general stress-response pathway (Fig. 3B) (Hengge-Aronis, 2002). Therefore, the phenotypes of *rpoS* and *rssB rpoS* mutants were analysed. The *rpoS* mutant exhibits a higher frequency of dead cells than WT, an inverse phenotype from the *rssB* mutant, consistent with the negative regulation exerted on RpoS by RssB. Furthermore, the double mutant also displays an elevated frequency of dead cells, indicating that the *rpoS* phenotype is epistatic to *rssB*, once again consistent with its downstream position in the pathway (Fig. 3C).

These results are consistent with the hypothesis that the phenotype of the *fliA* mutant is the consequence of its role in the motility regulon, and that the phenotype of the *rssB* mutant is mediated through the known role of RssB in the RpoS pathway.

A strain containing both mutants *fliA* and *rssB* also showed decreased cell mortality (Fig. 3), of the same magnitude as each of the single mutants, suggesting that the decrease of basal mortality in both mutants acts through a common mechanism or that there are epistatic interactions preventing additive effects in reducing death rates. The role of these two genes, and the implication of the phenotype of the double mutant (namely, that both mutants ultimately act through a common mechanism), are considered below.

### Potential cause of decreased dead cell frequency of *rssB* and *fliA* mutants

**Fig. 4:**
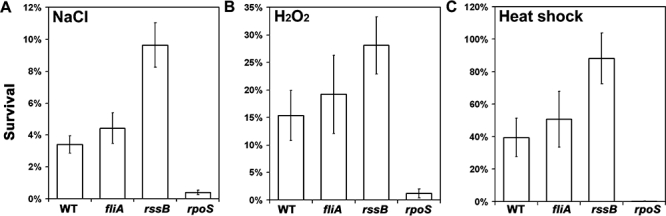
Resistance to external stress. Strains were tested for their resistance to (A) osmotic stress (2 M NaCl, 24 h), (B) oxidative stress (17 mM H_2_O_2_, 30 min), and (C) heat shock (55°C, 30 min). Error bars represent the standard error of the mean of six experiments.

One hypothesis to explain why these mutants are dying less is that they are more resistant to low-level stresses produced during growth. To test for generally elevated stress tolerance, the strains were measured for their resistance to exogenous stress such as oxidative stress, osmotic shock and heat shock. The *rssB* mutant, which results in elevated levels of RpoS (Hengge-Aronis, 2002), is more resistant to all of the tested stresses (oxidative and osmotic stress, both *P* < 0.005; heat shock *P* = 0.06; paired *t*-test), as would be expected from upregulation of the general stress response, while an *rpoS* mutant is much more sensitive than WT (Fig. 4). In the motility pathway, the *fliA* mutant (Fig. 4) is not significantly more resistant than WT to the stress conditions. While the results of the *rssB* mutant strain are consistent with the hypothesis of an elevated stress response increasing the ability of the cells to survive endogenous stresses, and thus die less frequently, it is not clear that this is the case in the motility pathway mutants.

To identify possible causes of the *fliA* mutant phenotype, several candidate genes were tested in an attempt to identify the downstream gene or genes responsible for the phenotype. These include *fliC*, the gene for the major structural protein of the flagellum, *flgJ*, encoding a muramidase responsible for insertion of the basal body of the flagellum through the cell wall, and *motA*, which lacks the motor proteins for turning the flagella (Macnab, 2003). The *fliC* mutant has no effect on mortality. The mutant *flgJ* has a reduced mortality in LB, which indicates that the insertion of the flagellum through the membrane may produce a local destabilization of the membrane, leading to death in some cells. The *motA* mutant exhibits a reduced ratio of dead cells relative to WT in both media (Fig. 3C). This mutant is likely to be phenotypically *motB* as well, due to polar effects within the operon and the reported instability of the MotB protein in the absence of its partner, MotA (Wilson and Macnab, 1990). These two motor proteins use a proton concentration gradient across the inner cell membrane (proton motive force; PMF) as an energy source for rotation of the flagellum (DeRosier, 1998). Therefore, the phenotype of the *motA* mutant may indicate that it is not only the physical structure of the flagellum which is responsible for cell mortality, but also the energy expenditure of the motor, that is at least partially responsible.

### Consequences of the mutants on cell fitness

**Fig. 5:**
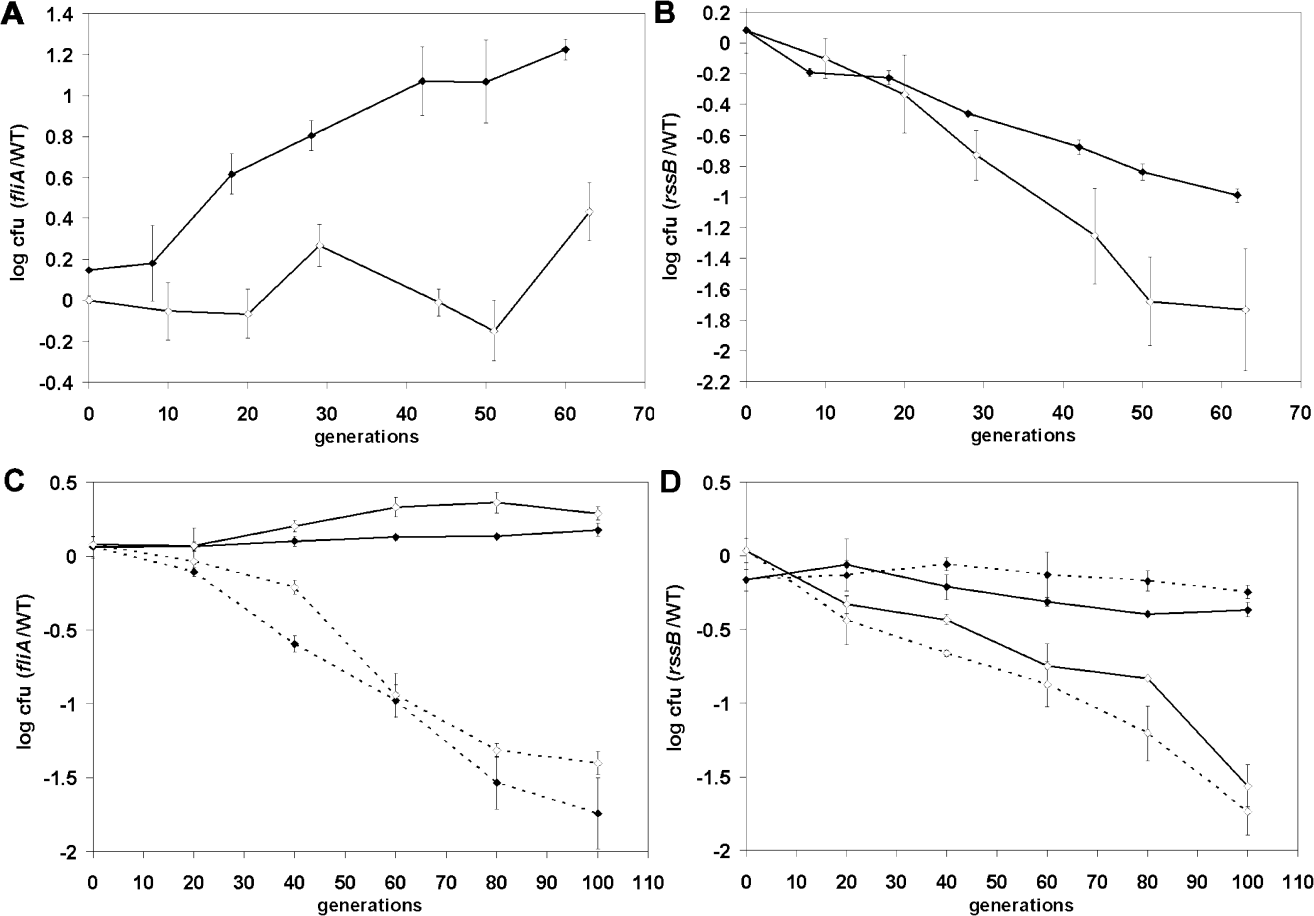
Competitions between mutants and WT. All competitions were performed in LB (solid symbols) and M9 (hollow symbols). A. The *fliA* mutant in liquid media with shaking. B. The *rssB* mutant in liquid media with shaking. C. The *fliA* mutant in structured media; dashed lines 3 g l^−1^ agar, solid lines 7.5 g l^−1^ agar. D. The *rssB* mutant in structured media; dashed lines 3 g l^−1^ agar, solid lines 7.5 g l^−1^ agar. All data have been log (base 10) transformed. The error bars represent the standard error of the mean of four experiments. All competition experiments were repeated with the markers swapped between strains; no significant differences were observed (data not shown).

In the absence of other effects, a population suffering fewer cell deaths per generation should have an advantage compared with the WT strain, and consequently, such strains should have been selected for under normal laboratory growth conditions. In order to determine whether these strains are, in fact, more fit, or whether there are associated phenotypes that adversely affect their ability to compete, they were grown in co-culture with WT in liquid M9 and LB cultures. The *rssB* mutant is out-competed by WT in both media, consistent with a cost coupled to the advantage of a lower frequency of dead cell production (Fig. 5B). Here, the cost may be a lowered growth rate due to the upregulation of the general stress response. Consistent with this, an *rpoS* mutant wins in competition against WT, presumably due to a reduced transcription of vegetative genes in the WT (King *et al*., 2004) (data not shown). In semisolid media, cell-to-cell competition is apparently reduced, probably due to spatial separation, although the *rssB* strain still may be somewhat disadvantaged (Fig. 5D).

The *fliA* mutant, on the other hand, has a clear competitive advantage over WT in LB, and no difference is observed in M9 (Fig. 5A). In fact, the advantage in LB is greater than that expected from the decreased dead cell frequency alone. From our results, the difference in dead cell frequencies alone should only account for a 2.5-fold difference in biomass between the *fliA* mutant and WT after 100 generations. This indicates that the strain has an additional growth advantage. In this case, the loss of the motility pathway seems to have improved cell fitness through both robustness and growth rate. The obvious cost associated with these mutants, the loss of motility, apparently does not play a role in liquid culture with vigorous shaking. The competitions performed under the alternative laboratory conditions of semisolid and solid agar plates, however, do not show this advantage. Under these conditions, when the agar percentage is low enough to allow cell motility, the *fliA* mutant is rapidly out-competed by WT (Fig. 5C). At higher percentages of agar, the strains are nearly equivalent, again probably due to reduced competition. These results – indicating that in solid media there is no selection against loss of motility, and in semisolid conditions there is selection for it – may help explain the continued motility of at least some WT strains under laboratory conditions.

### Sigma factor balance

That both of these mutants directly affect the level of sigma factors within the cell may be significant in explaining the general mechanism by which an alteration in growth rates and death frequencies is achieved. Indeed, sigma factors have been proposed to compete in the cell for free RNA polymerase (Ishihama, 2000; Jishage *et al*., 2002; Nystrom, 2004). The *rssB* mutant clearly alters the balance of sigma factors within the cell, increasing the stress-response sigma factor levels (Zhou and Gottesman, 1998), thereby increasing stress resistance. In the case of *fliA*, the loss of the motility sigma factor (calculated to occupy 14% of the RNA polymerase in the cell; Ishihama, 2000) is expected to result in greater resource availability, both in the form of free RNA polymerase and in energy saved through the lack of a drain on the PMF. Contrary to *rssB*, in which we observe threefold more σ^S^ and twofold less σ^70^ in early stationary phase than in the WT strain, there is no difference in the protein level in the *fliA* strain (data not shown). To test specific sigma factor activities, a strain containing three orthogonal fluorescent reporter proteins under the control of different regulons within the cell was constructed. This stress-reporting (SR) strain expresses a modified cyan fluorescent protein (CFP^++^) from the *rrnB* P2 promoter under the control of σ^70^, the main vegetative growth sigma factor in the cell (Murray *et al*., 2003). A modified yellow fluorescent protein (YFP^++^) reporter is expressed from the *yiaG* promoter under the control of σ^S^ (RpoS) (Shimada *et al*., 2004; and C. Saint-Ruf, pers. comm.), and red fluorescent protein (mRFP1; Campbell *et al*., 2002) is expressed from the *ibpAB* promoter, under the control of σ^32^, the heat-shock sigma factor (Laskowska *et al*., 1996; Kuczynska-Wisnik *et al*., 2002). These reporters are expressed under different stress conditions known to activate expression of genes under σ^S^ and σ^32^ (10- and 3-fold induction respectively in stationary phase and in acidic shock for *yiaG:*:YFP; 4- and 3.5-fold respectively in heat shock and ethanol shock for *ibpA*::RFP) (data not shown).

**Fig. 6:**
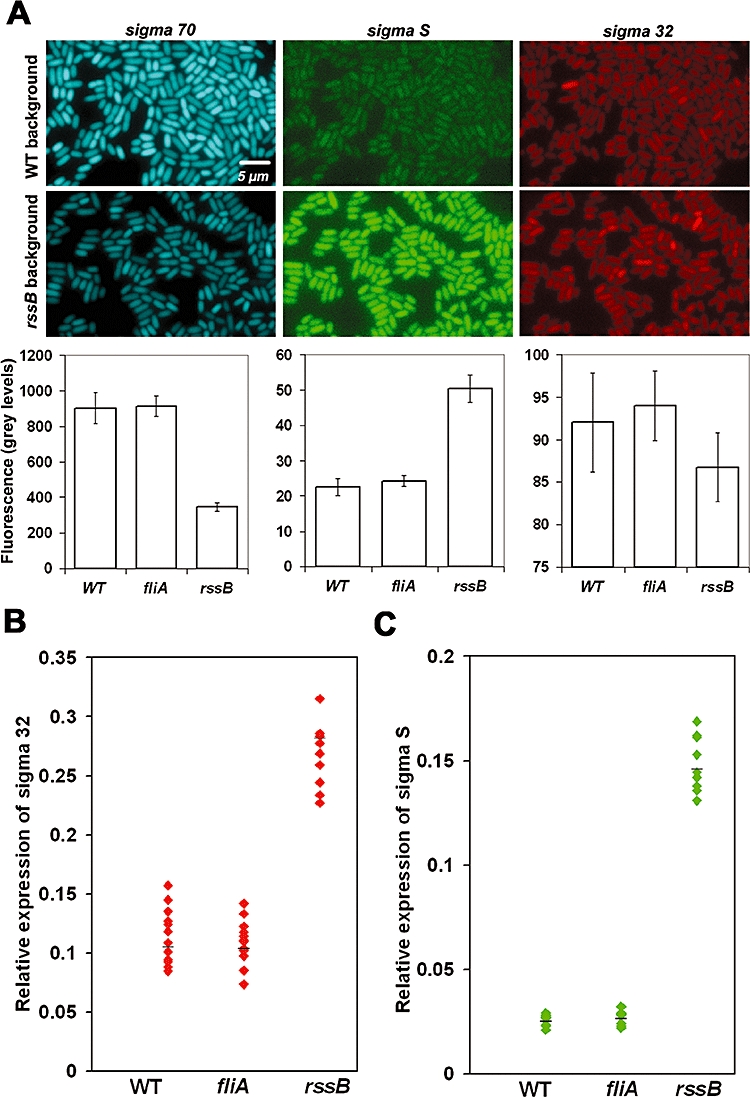
Relative gene expression levels controlled by three σ factors. A. Images of one field of strains carrying fluorescent reporter proteins under the control of promoters responsive to σ^70^ (blue, left), σ^S^ (green, middle) and σ^32^ (red, right). Each graph corresponds to the mean fluorescence intensity for each reporter. B. Normalized expression from the σ^32^ reporter in the mutant strains and WT. This reporter is significantly different from WT in the *rssB* strain (*P* = 0.007), but not in the *fliA* strain (*P* = 0.344), by unpaired data *t*-test. C. Normalized expression from the σ^S^ reporter in the mutant strains and WT. Both strains are significantly different from WT for this reporter (*rssB*, *P* < 0.001; *fliA*, *P* = 0.007) by unpaired data *t*-test. Each point represents one experiment; the black bar represents the mean of each strain.

Both the *fliA-* and *rssB*-null mutants were analysed in the SR strain background, and compared with the WT SR strain. Fluorescence levels were measured in > 10^5^ single cells in each strain through the semiautomated analysis of microscope images (Fig. 6A). As expected, the *rssB* strain shows a higher absolute level of expression from an RpoS-dependent promoter, but no significant difference in the absolute level of the heat shock expression as compared with WT (Fig. 6A). As the absolute level of expression from the vegetative growth reporter is lower compared with the WT strain, there is a relative increase in the levels of both stress reporters in the *rssB* strain (Figs 6B and 5C), which is consistent with the increased stress resistance exhibited by the strain, as well as its reduced growth rate.

Comparing the *fliA* strain with WT, there is a much smaller difference in the absolute levels of the three reporters (Fig. 6A). Relative to the general growth reporter, however, there is a small but significant difference in the level of the RpoS reporter (Fig. 6C). As the complete loss of the motility pathway sigma factor has not greatly affected the balance between the three measured sigma factors, these results reinforce the hypothesis that the increased viability in this strain is due to the loss of a cost associated with motility, such as a reduced membrane destabilization and a reduced drain on the PMF.

## Discussion

From a screen designed to identify mutants with altered cell death frequencies, we have identified two mutants, *rssB* and *fliA*, that exhibit reduced quantities of dead cells. Dead cells were measured by two criteria: presence of a destabilized membrane, and an incapacity to divide. These two approaches report different categories of cells that can be considered dead, and they yield similar results. We conclude that the products of these two genes, in the WT strain, are involved in processes that directly or indirectly increase the probability of death of at least a fraction of the individuals in a population.

As this decreased death frequency manifests in the apparent absence of exogenous stress, it is probable that the original causes of death arise from endogenous events in the cell, and that the absence of these genes results in either the reduced occurrence of these internal events, or an improved ability to survive them. The striking similarity between these mutations is that each has an immediate effect on the level of one of the sigma factors (the subunit of RNA polymerase responsible for determining the global classes of genes transcribed) within the cell. Beyond this similarity of gene class, the two gene products are thought to operate on different pathways. However, the double mutant strain (*fliA rssB*) does not exhibit a lower mortality than either of the mutants separately. One possible explanation for this observation is that there is an ultimate, common mechanism, through which both mutants act (possible candidates for such a function are discussed below); alternatively, epistatic interactions may prevent additive effects.

The loss of the *rssB* gene product results in the stabilization of RpoS, and therefore increased levels of this stress-response sigma factor. The general stress-response system is thus expressed at a higher level than in WT, as shown by elevated levels of the RpoS-driven fluorescent reporter. The mutant strain is subsequently more resistant to a variety of exogenous stresses, and hence is likely to be better able to respond to the occasional endogenous stress that otherwise could potentially prove fatal. In the WT strain, such events may either act too quickly for the stress system to be induced, or fail to induce the system at all. The *rssB* mutation shows that it is possible for the cell to increase its robustness, which, in the absence of other pleiotropic effects, would increase the fitness of the cell. That such a mutation has not already become fixed in the population during the history of the strain growing under laboratory conditions may imply that such an alteration carries an associated cost of at least equal magnitude. By competing the mutant against the WT strain in serial batch culture, we have demonstrated that the mutant does exhibit a globally reduced fitness compared with the parental strain. This loss of fitness apparently manifests through a reduced growth rate, possibly caused by the reduced absolute levels of expression from the general growth reporter, as seen in the SR fluorescent strain (Fig. 6A). Consistent with this hypothesis, the loss of the stress-response sigma factor RpoS, while resulting in elevated cell mortality, also results in increased fitness in competition with WT. Such mutants of *rpoS*, originally identified for their GASP (growth advantage in stationary phase) phenotype, have been found to be selected under certain conditions of laboratory growth, such as nutrient-limited conditions and stationary phase (Zambrano and Kolter, 1996; Vulic and Kolter, 2001), indicating that selection for growth over maintenance is readily possible (Notley-McRobb *et al*., 2002; Ferenci, 2005). For most populations of the laboratory strain, however, the evolutionary pressures on these life-history traits results in levels of repair and growth investment (provided by functional RssB and RpoS proteins) that are less than maximal, but apparently a good compromise for the different environments the cells experience.

The inactivation of the *fliA* gene, encoding the flagellar sigma factor σ^F^, results in the lack of expression of a number of genes involved in motility and chemotaxis, and consequently, non-motile cells (Liu and Matsumura, 1995). This loss of motility should result in greater absolute availability of both RNA polymerase and energy for other processes within the cell. Unlike the *rssB* strain, the results from the fluorescent stress reporter strain show only modest alterations in the balance between growth and maintenance. While not significantly more resistant to external stress, the *fliA* strain does exhibit a greater relative fitness than WT in competition in liquid culture in rich media. This advantage is greater than the expected benefit from having fewer dead cells, indicating that the strain possesses multiple advantages over WT under these conditions. The obvious cost associated with this mutation, the loss of motility, is clearly unimportant under these conditions of equal access to nutrients due to the shaking of the culture during incubation. On solid agar media (7.5 g l^−1^), where the cells are prevented from swimming whether they are motile or not, the *fliA* strain again shows no cost associated with the mutation, although the benefit is reduced compared with liquid media, probably due to the restrictions on competition inherent in being fixed in place. It is only in semisolid agar (3 g l^−1^) that the cost of the loss of motility becomes apparent. Under these conditions, cells that are motile can use chemotaxis to acquire nutrients, as opposed to waiting for their arrival by diffusion. The advantage is strong enough to completely reverse the balance with WT compared with liquid media, indicating the extremely useful nature of motility and chemotaxis under such conditions. For laboratory strains, it appears from these experiments that the common shaken liquid culture may select for non-motile strains, where the loss of FliA represents an adaptation without cost, while solid agar plates and stabs are approximately neutral. This observation helps explain the earlier finding that some (2 of 11) laboratory strains of *E. coli* have lost motility through the loss of the FliA sigma factor during laboratory passage and storage (Jishage and Ishihama, 1997). In cultures from long-term evolution experiments, it was also found that several flagellum operons exhibited decreased expression (Cooper *et al*., 2003).

By losing the motility pathway, there are a number of potential mechanisms by which the cell may gain viability. First, the synthesis of the flagellum itself is costly, and by not producing it, the cell could use those resources elsewhere. Our finding that the loss of *fliC*, the gene encoding the major subunit of the flagellum, does not significantly affect cell death frequency, makes this explanation difficult to support. Second, the physical penetration of the flagellum through the cell envelope may weaken cells, decreasing their robustness. Supporting this, a strain lacking the muramidase (FlgJ) necessary for opening the cell wall to permit the extension of the flagellum to the exterior of the cell shows decreased mortality in rich media. Third, by not powering the flagellar motor, there is a reduced energy drain on the PMF. This could result in two possible benefits; the energy saved could be used elsewhere in the cell, or there may be reduced internal stress due to this absence of energy use. Consistent with this latter mechanism, a strain lacking the motor proteins does show a significant reduction in mortality. Therefore, the most likely sources of reduced mortality in this mutant are increased energy availability, or more probably, a decreased internal cell stress due to the absence of physical destabilization of the membrane and the absence of energy use by the motor. To place the level of energy use in context, the quantity of proton use per cell, per second, for rotation of the flagella is equivalent to the number of protons used to produce 3.2 × 10^5^ ATP by the F0F1 ATPase, and the cost of the flagellum rotation corresponds to 0.1% of the cell's total energy expenditure (Harold and Maloney, 1996; Macnab, 1996).

The fact that the double mutant, *fliA rssB*, does not show an increased effect relative to either single mutant leads to the possibility that both mutations act through a common mechanism at some point (alternatively, epistatic interactions may prevent additive effects). While we cannot exclude the possibility that both mutations increase the level of σ^S^-RNA polymerase complex available for an unidentified maintenance or repair pathway, this pathway would also have to be saturated by either mutant alone, in order to explain the phenotype of the double mutant. Alternatively, it may be more likely that the flagellar motor causes endogenous stress within the cell, either directly or indirectly, and that the two mutants, *rssB* and *fliA*, both act through the reduction of this stress; specifically, *rssB* by elevated levels of the general stress response, and *fliA* by elimination of the flagellum structure and motor. Such an explanation is consistent with the phenotype of the double mutant, and the known roles of the two gene products.

We conclude that selection on WT cells does not favour the maximized extremes of robustness or growth rate, but rather that there is a trade-off between these and other traits, and that the current performance of the cell is a compromise among them. That such trade-offs exist is well established by ecological studies in different organisms (Stearns, 1989). Here we present, in the well-characterized model organism *E. coli*, the possibility of identifying and understanding the molecular mechanisms and interactions behind such internal competitions for limited resources.

## Experimental procedures

### Strain construction

All strains are derived from the sequenced WT strain of *E. coli* MG1655 (Blattner *et al*., 1997). To construct the library, the WT strain MG1655 was randomly mutagenized by Tn10 insertion, using a modified lambda vector λNK1323 (Kleckner *et al*., 1991). A total of 3000 mutants were isolated in M9 minimal media with 0.4% glucose at 30°C, were purified, incubated, cultivated and individually conserved in 20% glycerol in 96-well microplates (ABgene) at −80°C. Each microplate contains eight uninoculated wells as controls for contamination, and two wells contain strains with a Tn10 insertion in the *lacI* gene, used as WT controls for screening experiments. Strains used for all experiment are described in Table S2. Deletions were constructed by replacing the entire deleted ORF from start to stop codon with a chloramphenicol (Cm) antibiotic cassette, which was then removed by FRT recombination using the method of Datsenko and Wanner (2000) and confirmed by PCR. These deletion strains with antibiotic markers removed were used for all experiments subsequent to the screen. Primers used to create the deletions are listed in Table S3. The strains used for competition experiments, with resistance to nalidixic acid and streptomycin, or with mRFP1 or YFP under the control of a Ptet promoter, were made by P1vir transduction.

For the SR strain, with fluorescent reporters for σ^70^, σ^32^ and σ^S^, the three reporter constructs were introduced successively into the original WT strain. For σ^70^, CFP was placed under the P2*rrnB* promoter in an IS sequence (Murray *et al*., 2003). For σ^32^, mRFP1 was put under the control of the *ibpAB* promoter in the *intC* sequence. For σ^S^, YFP was placed under the promoter of the RpoS-dependant gene *yiaG* at the native position of the gene in the chromosome. A complete description of this strain will be published by one of the authors (A.B.L.). Finally, deletions of *fliA* and *rssB* were introduced into the SR strain by P1 transduction.

### Analysis of the mutants

To isolate candidates with altered proportions of dead cells, cultures of mixed mutants (see above) were grown in 96-well microplates with 200 μl of M9 + 0.4% glucose + tetracycline at 30°C, with shaking, overnight, then spotted onto dilute LB agar plates (yeast extract 0.1 g l^−1^, tryptone 0.2 g l^−1^ Difco, NaCl 10 g l^−1^, Acros) plus 0.03% PI (Molecular Probes), without tetracycline, and incubated overnight (15 h) at 42°C. Plates were screened under a fluorescence microscope (Zeiss 200 M; Zeiss, Jena, Germany). Grey-scale images were taken with a CoolSNAP HQ (Princeton Instruments, Trenton, NJ, USA) at 40× magnification.

Quantitative data on the proportion of dead cells were produced by FC. Cells were grown in filtered (0.2 μm filter) LB or M9 at 30°C. Cultures were diluted in 500 μl of filtered M9, 3 mM MgSO_4_, and 0.5 μM SYTOX Green (SG; Molecular Probes), to approximately 5 × 10^6^ cfu ml^−1^, then incubated for 30 min at room temperature in the dark. In total, 10^6^ cells were counted per culture in a BDLSR2 flow cytometer (Beckton Dickinson) with a 488 nm laser. All parameters were collected as logarithmic signals. Samples were run such that the event rate was 3000 events per second, at the high flow rate settings. Green fluorescence from stained cells was collected in the FL1 channel (530 ± 15 nm). Dead cells with compromised membranes allow penetration of SG, as PI. SG is used in FC, as it is known to give a greater enhancement (100-fold) in fluorescence intensity on staining-compromised, as opposed to healthy, cells compared with PI (Mortimer *et al*., 2000). The two populations are easily separable, and the proportion of dead cells was calculated as a percentage for each strain.

The M9 cultures analysed by FC were also tested for viability by the incapacity of cells to grow and form a microcolony. For this, 2 μl of a culture diluted 1:2000 was spread on a microscope slide covered with M9 + 0.4% glucose agarose. Slides were incubated at 30°C for 4 h. The single cells and cells that formed microcolonies were then counted.

To identify the mutation responsible for the phenotype in each isolate, P1 stocks were made on mutant candidates and transduced into the WT strain. One hundred transductants were tested for the observed phenotype, giving the percentage of co-transduction between the Tn10 insertion (tetracycline resistance) and the observed phenotype. The mutants tested have a greater than 98% frequency of co-transduction. The DNA sequence flanking the transposon insertion was determined by a PCR-based protocol using arbitrary-primed PCR (Caetano-Anolles, 1993). The first round of amplification was performed with two arbitrary primers, ARB1 and ARB6, and a third primer specific for the Tn10 transposon. The second round was performed on the product of the first PCR step, using ARB2 and a primer complementary to a sequence 20–30 bp from the end of the Tn10 transposon (see Table S3 for primer sequences). The PCR product was purified with the Qiagen PCR purification system, sequenced and compared with DNA sequence databases.

### Competitions

For liquid-media competitions involving the *rssB* strain, and the *fliA* strain in LB, antibiotic resistance markers for streptomycin and nalidixic acid were used. In the case of *fliA* competitions in liquid M9, and all competitions in structured media, fluorescent markers (mRFP1 and GFP) were used, under the control of the Ptet promoter. For all liquid-culture competitions, WT and mutant strains were diluted at a 1:1 ratio in 10 ml LB or M9 (0.4% glucose) and were incubated at 30°C. In LB, the culture was diluted 1000-fold, three times per day at OD_600_ = 0.8–1.0, and in the morning when the culture is saturated (OD_600_ = 2). In M9, the cultures were diluted 1000-fold, two times per day, every 12 h at OD_600_ = 1. For enumeration of the antibiotic-marked strains, the number of cfu of both strains was counted by plating cells on LB plates containing nalidixic acid (40 μg ml^−1^) or streptomycin (25 μg ml^−1^). Fluorescent bacterial counts were made on LB plates with 50 μM aTet (Acros) after 48 h incubation at 37°C using a tunable lighting system (LT-9500–220 illumatool, Lightools Research, Encinitas CA, USA). Colonies producing fluorescent protein were detected using 470 ± 40 or 540 ± 10 nm wavelength excitation light and a 515 or 590 nm reading filter for GFP and mRFP1 respectively.

For competition in structured media, all strains were introduced at a 1:1 ratio into 4 ml of molten (50°C) agar (LB with 0.3% or 0.75% agar, Difco). The agar suspension was then mixed and spread on Petri plates containing 25 ml of hardened bottom agar (1.5% agar in M9 with 3 mM MgSO_4_). After an incubation of 24 h at 30°C, the soft agar matrix was scraped into 10 ml of 0.01 M MgSO_4_, and shaken vigorously for 5 min to free the bacteria. Fluorescent bacterial counts were made as described above. Successive serial transfer cycles were initiated by adding approximately 10^4^ cells into 4 ml of fresh agar.

In order to control for marker effect, all competition experiments were repeated with the markers swapped between strains.

### Resistance to external stress

Assays were conducted on exponentially growing cells (OD_600_ between 0.15 and 0.30) cultivated in minimal media (M9 + 0.4% glucose). For oxidative stress, cells were exposed to 17.6 mM H_2_O_2_ (Sigma) added to the medium during 30 min, before inactivation by catalase (Roche, 2 μg ml^−1^). For heat shock, 500 μl of cultures was incubated with shaking at 55°C during 30 min, in 12-well microplates (Greiner). Osmotic stress was performed by adding NaCl (2 M final) to cultures incubated for 24 h. The percentage of survivors after exposure to the stress conditions was measured by plating the bacteria directly onto LB plates and counting colonies after overnight incubation at 37°C.

### Single-cell fluorescence analysis

Cultures of the SR strain expressing three fluorescent reporters were concentrated and spread on agarose without carbon source (Qbiogene) in a cavity slide to obtain a cell monolayer, as described previously (Stewart *et al*., 2005). A series of images were recorded at 100× magnification, in phase contrast and in fluorescence [50% neutral density filter on a 100 W Fluo-Arc Hg-vapor lamp (Zeiss) regulated to 50% power] at wavelengths of 500 nm (YFP), 540 nm (mRFP1) and 420 nm (CFP) during respectively 6, 6 and 4 s of exposure time. Images were analysed using Metamorph software (Meta Imaging Systems). Cells were identified as objects in the phase-contrast image, and a mask of the cell outlines was applied to each fluorescence image. On average, 100 000 cells were analysed per strain. The fluorescence background of the agarose was recorded and subtracted from each cell value. For each cell, we obtained the average intensity per pixel for each fluorescent protein. Statistical *t*-tests between strains were made on the average values of each reporter.

## Supplementary material

This material is available as part of the online article from:

http://www.blackwell-synergy.com/doi/abs/10.1111/j.1365-2958.2007.05988.x

(This link will take you to the article abstract).
