# Supplementary material for: Mutations in two global regulators lower individual mortality in Escherichia coli
Source: Mol Microbiol. 2007 Nov 25;67(1):2–14. doi: 10.1111/j.1365-2958.2007.05988.x (PMC2229837; doi:10.1111/j.1365-2958.2007.05988.x)
Supplement: Supplementary file 1 [file mmi0067-0002-SD1.pdf]

Table S1: Mutants isolated from the qualitative screen. Ten mutants were isolated and identified as having less dead cells than WT on solid media. The mutant name represents the position of the mutant in the ordered library. Tn10 insertion locations were determined by arbitrary PCR and sequencing (see materials and methods). The functions of the genes affected by the Tn10 insertion are from the SWISSPROT database. ND= not determined.

**Mutant name**	**Tn10 insertion (location)**	**Function of gene affected by Tn10 insertion**	**Ratio percentage of dead cells WT/mutant In LB 30°C**	**Ratio percentage of dead cells WT/mutant in MM 30°C**
44D3	*rssB* *27'79*	negative regulator of σ^S	3.3 (+/- 0.03)	3.7 (+/- 0.04)
46E3	*znuC, znuB41'84*	high affinity ABC transport system for Zn	ND	ND
47B3	*yiaT* *80'81*	potential outer membrane protein (similar to *mipA* family)	ND	ND
47E4	*b2881* *65'08*	unknown function	1.64 (+/- 0.04)	1.90 (+/- 0.03)
48H10	*yhaB* *70'39*	unknown function	1.12 (+/- 0.02)	1.95 (+/- 0.14)
59H8	*yieC* *84'03*	potential outer membrane protein (similar to *lamB* family)	0.92 (+/- 0.02)	1.49 (+/- 0.07)
65D2	*yjdK* *93'77*	unknown function	2.21 (+/- 0.03)	1.88 (+/- 0.15)
64D10	*yfdV* *53.61*	potential inner membrane protein	1.08 (+/- 0.08)	1.27 (+/ 0.15)
66G11	*ykgG* *6'*	unknown function	1.32 (+/- 0.06)	1.3 (+/- 0.3)
70B5	*fliA* *43'09*	σ^F (motility and flagellar synthesis)	2.5 (+/- 0.04)	6.5 (+/- 0.02)

Table S2: Stains used in this study. All strains are derived from *E. coli* K-12 (MG1655).

Strain name	Relevant genotype	Source/ reference
**NEC283**	*ΔfliA FRT*	This work
**NEC284**	*ΔflhD FRT*	This work
**NEC285**	*ΔflgM FRT*	This work
**NEC286**	*ΔfliC FRT*	This work
**NEC287**	*ΔflgJ FRT*	Allele from Keio collection
**NEC288**	*ΔmotA FRT*	Allele from Keio collection
**NEC289**	*motA448 zea ::*Tn10	(Samuel and Berg, 1996)
**NEC290**	*ΔrssB FRT*	This work
**NEC291**	*rpoS : :*Tn10	(Bjedov *et al.*, 2003)
**NEC292**	*ΔrssB rpoS : :*Tn10	This work
**NEC293**	*ΔfliA FRT ΔrssB FRT*	This work
**NEC294**	*rpsL(StrepR)*	(Giraud *et al.*, 2001)
**NEC295**	*ΔfliA rpsL*	This work
**NEC296**	*ΔrssB rpsL*	This work
**NEC297**	*gyrA (nalR)*	(Giraud *et al.*, 2001)
**NEC298**	*ΔfliA FRT gryA*	This work
**NEC299**	*ΔrssBFRT gyrA*	This work
**NEC300**	*ypdA*::*Patet*-GFP^{++}	This work
**NEC301**	*ΔfliA ypdA*::*Patet*-GFP^{++}	This work
**NEC302**	*ΔrssB ypdA*::*Patet*-GFP^{++}	This work
**NEC303**	*Patet-m*RFP	This work
**NEC304**	*ΔfliA ypdA*::*Patet-m*RFP	This work
**NEC305**	*ΔrssB ypdA*:: *Patet-m*RFP	This work
**NEC306**	*PibpAB-m*RFP *; intC ::P2rrnB*-CFP^{++} *; PyiaG*-YFP^{++}	This work
**NEC307**	*ΔrssB PibpAB-m*RFP *; intC ::P2rrnB*-CFP^{++} *; PyiaG*-YFP^{++}	This work
**NEC308**	*ΔfliA PibpAB-m*RFP *; intC-P2rrnB ::*CFP^{++} *; PyiaG*-YFP^{++}	This work

Table S3: Primers used in this study. Underlined portions of the primers for deletion correspond to the sequence for Cm cassette amplification.

**Primer name**	**Sequences (5'-3')**
rssB delfor	atgacgcagccattggtcggaaaacagattctcattgtt ggtgtaggctggagctgcttc
rssB delback	tcattctgcagacaacatcaagcgcagtcgaccaccggtt catatgaatatcctccttag
rssB checkfor	gcagtcgttaacccaatttc
rssB checkback	ggcaacatcctggttctta
fliA delfor	actcctggtagtcaaagttaaagtgcggcatttactgacg gtgtaggctggagctgcttc
fliA delback	taatcatgccgataactcatataacgcagggctgttttatc catatgaatatcctccttag
fliA checkfor	aacaactccggctacatctt
fliA checkback	gcttatcaggcctacaagt
flgM delfor	cagttactctgcaagtcttgctgcgcttcgttgatcagcgcatcggc gtgtaggctggagctgcttc
flgM delback	gagtattgatcgcacttcgcctctgaagcctgtaagcaccgttcaaccgc catatgaatatcctccttag
flgM checkfor	tgaacctggcgtttattggtc
flgM checkback	gcgctaaggctcatctggtat
flhD delfor	caggcccttttcttgcgcagcgcttcttcaggctgattaacatc gtgtaggctggagctgcttc
flhD delback	gtgggaataatgcatacctccgagttgctgaaacac catatgaatatcctccttag
flhD checkfor	caaacgccggaagtgagtc
*flhD*checkback	caacccggtacgcaccaga
fliC up	atggcacaagtcattaataccaacagcctctcgctgatca tgtgtaggctggagctgcttc
fliC dwn	ttaaccctgcagcagagacagaacctgctgcggtacctgg catatgaatatcctccttag
fliC checkfor	cctgacccgactcccagc
fliC checkback	tcggacgattagtgggtg
ARB1	ggccacgcgtcgactagttacNNNNNNNNNNgatat
ARB2	ggccacgcgtcgacttagttac
ARB6	ggccacgcgtcgactagtacNNNNNNNNNNacgcc
Tn10ext	tggaaggaacgtcaattccc
Tn10int	ccattgctgttgacaaaggg

References

Bjedov, I., Tenaillon, O., Gerard, B., Souza, V., Denamur, E., Radman, M., Taddei, F., and Matic, I. (2003) Stress-induced mutagenesis in bacteria. Science **300**: 1404-1409.

Giraud, A., Matic, I., Tenaillon, O., Clara, A., Radman, M., Fons, M., and Taddei, F. (2001) Costs and benefits of high mutation rates: adaptive evolution of bacteria in the mouse gut. Science **291**: 2606-2608.

Samuel, A.D., and Berg, H.C. (1996) Torque-generating units of the bacterial flagellar motor step independently. Biophys J **71**: 918-923.
